# Multiple molecular characteristics of circulating enterovirus types among paediatric hand, foot and mouth disease patients after EV71 vaccination campaign in Wuxi, China

**DOI:** 10.1017/S0950268822000784

**Published:** 2022-04-27

**Authors:** Yan-Jun Kang, Chao Shi, Jian Zhou, Jun Qian, Yuanwang Qiu, Guizhi Ge

**Affiliations:** 1 Department of Pediatric Laboratory, Wuxi Children's Hospital, Wuxi, China; 2 Department of Disease Control, Wuxi Center for Disease Control and Prevention, Wuxi, China; 3 Department of Pediatrics, Wuxi Children's Hospital, Wuxi 214023, China; 4 Department of Infectious Diseases, The Fifth People's Hospital of Wuxi, Wuxi, China; 5 Department of Infectious Disease, Wuxi Children's Hospital, Wuxi, China

**Keywords:** Hand, foot and mouth disease, Molecular characteristics, Enteroviruses, EV71 vaccination, Wuxi

## Abstract

The molecular properties of the circulating causative agents of hand, foot and mouth disease (HFMD) in Wuxi remain unclear, posing diagnostic and prevention challenges. Additionally, in several regions of mainland China, the EV71 immunisation drastically reduced related cases and altered the HFMD pathogen spectrum, while the precise situation in Wuxi remained unknown. To address these issues, paediatric HFMD cases diagnosed in the clinic were enrolled and anal swabs were acquired in the spring of 2019. The 5′-UTR and VP1 genes were interpreted using RT-nPCR with degenerate primers to confirm their genotypes. Following that, the entire genome sequences of each viral type were recovered, allowing for the interpretation of several molecular properties. A total of 249 clinically confirmed HFMD cases had their anal swabs taken for viral identification, from which the genome sequences of seven genotypes were recovered. Coxsackievirus A16 is the most prevalent type, followed by Coxsackievirus A6, A10, A2, A4, A5 and Echovirus 11, all of which were genetically determined for the first time in Wuxi. Phylogenetic and recombination analyses were used to evaluate their evolutionary relationships with other strains found in other regions. Noticeably, a CVA16 subtype, responsible for a large proportion of the observed cases, phylogenetically clustered within clade B1a along with some strains from other countries, was the first one to be reported in China. Furthermore, some recombination events were inferred from strains detected in sporadic cases, particularly the recombination between CVA2 and CVA5 strains. Our investigation elucidated the multiple molecular characteristics of the HFMD causal enterovirus strains in Wuxi, underlining the potential hazards associated with these circulating viral types in the population and aiding in future surveillance and prevention of this disease.

## Introduction

Hand, foot and mouth disease (HFMD) is a childhood infectious disease that is mostly caused by many enteroviruses (EVs), such as Coxsackievirus A16 (CVA16), Coxsackievirus A6 (CVA6), Coxsackievirus A10 (CVA10) and Enterovirus 71 (EV71) [1, 2]. While this condition is often harmless and self-limiting, the high transmission rate of EVs leads to a huge number of infected cases worldwide, particularly in the Asia-Pacific area [2]. As a result, the number of severe or fatal cases of HFMD was substantial. To date, HFMD remains a major public health concern due to the high illness burden it has caused in many countries, including China [3].

In China, the most prevalent EV serotypes responsible for HFMD were EV71 and CA16, with EV71 responsible for 70% of severe cases and 92% of deaths [4, 5]. Due to this, EV71 was the primary focus of disease prevention in China during the past two decades. Hence several monovalent, inactivated whole-virus vaccines against EV71 have been successfully developed, and a vaccination programme for children has been gradually implemented in many regions of China since 2016. This has helped stop the spread and prevalence of EV71. In recent years, it is believed that the EV71 has been supplanted by some other EV types, such as the CVA16 and CVA6, as the primary causes of HFMD in a number of locations [5–7]. Additionally, several other circulating EV strains, whether associated with severe cases or not, have altered or been replaced significantly over time in response to different preventative strategies [8].

As an important city in the Yangtze River Delta region of China, the HFMD epidemic in Wuxi should be taken seriously, as surveillance data show that the incidence of HFMD in the region is higher than in the surrounding areas [9]. But the knowledge accumulation of HFMD in this region was restricted to the preliminary epidemiology studies concerning the basic statistical data and circulating serotypes of EVs [9, 10]. In particular, considering the clinical meaning, some studies were restricted to the pathogens of HFMD severe cases. Some epidemiological investigations, on the other hand, used quick tests to reveal the basic serotype information of circulating predominant EV strains. Generally, before 2018, the HFMD associated pathogens were dominated by EV71 (46.08%) and CA16 (35.78%), along with CB5 (72.50%) in the ‘other’ (Non-CVA16 and Non-EV71) group [10]. To date, the molecular properties of the circulating viral types have not been characterised, which has hampered the diagnosis and prevention of HFMD in Wuxi. In addition, the situation of the EV71 vaccination impact was unclear, even though it is of high reference value for the prediction of the following predominant genetic types and epidemics.

To address this issue, we collected more than 200 clinically diagnosed HFMD outpatients and pursued the genetic information using degenerate RT nested PCR, and recovered their genome sequences. Based on data from the HFMD surveillance system, the dynamics of the pathogen shift of HFMD in Wuxi over the last decade were deciphered. The results revealed EVs of high diversity associated with HFMD and, compared to the existing strains, some of them were novel or first reported in Wuxi. Besides, the whole genomes of almost all viral strains were recovered and their phylogenetic and recombination features were disentangled.

## Materials and methods

### Patients and data collection

The sample collection took place in the spring of 2019 and lasted for a total of three months. The ethics committee of Wuxi Children's Hospital gave its clearance for this research. All participants or their guardians were notified and consented to the collection prior to the enrolment.

We focused on paediatric patients with suspected HFMD symptoms who were admitted to the department of infectious disease of the hospital throughout this time period. We defined a probable HFMD patient as a patient who had a papular or vesicular rash on their hands, feet, mouth, or buttocks, with or without fever [2]. Then, the recruitment criteria were: participants who had certain clinical indications, such as skin eruptions on their hands and feet, and vesicles in their mouths, were randomly chosen. The stool samples from these chosen children were obtained using anal swabs with Universal Transport Medium (UTM), and they were promptly sent to the −80 °C storage facility for further investigation. The epidemiological data for 2010–2019 was obtained from the Chinese national HFMD surveillance system via the Wuxi Center for Disease Control and Prevention, which included information on the number of cases, the incidence rate and the pathogen responsible.

### Determination of the genotypes and whole-genome sequences

The total RNA was extracted using the RNeasy Plus Universal Mini Kit (QIAGEN) according to the manufacturer's instructions. The initial genetic detection, which is intended to determine fundamental serotype information, was carried out using nested reverse transcription PCR (RT-nPCR) based on generic degenerate primers targeting the 5′-UTR region of three EV species, as reported [11]. The PCR-generated products were purified by agarose gel electrophoresis before being directly resolved using Sanger sequencing. If sequencing failed, the fragment was then cloned into the pMD18-T vector and multiplied in *Escherichia coli* (strain DH5*α*) for further sequencing. Following that, primers targeting the VP1 genes of three EV groups were used to validate their precise genetic types. After the genotype information was determined, one strain from each of the identical kinds was chosen to pursue its whole genome using the degenerate primers designed based on the conserved regions of the genome alignment with reference strains. The acquired sequences from the same viral strain were assembled by using the Seqman software in the Lasergene packages. The open reading frames (ORF) of the genome sequence were identified using the online programme ORFfinder (https://www.ncbi.nlm.nih.gov/orffinder/) if the entire genome sequences were successfully recovered. After that, the assembled sequences were searched using BLASTn against the NCBI nucleotide (nt) database via the online Blast programme (https://blast.ncbi.nlm.nih.gov/Blast.cgi) of NCBI. In addition, the precise sites of various coding regions in the genome were determined with reference to the prototype of each EV type.

### Phylogenetic and recombination analysis

To infer the phylogenetic traits of the detected viral strains along with other closely related viruses or those out of the same taxonomic status, we aligned these sequences by using MEGA7 software, deploying the MUSCLE algorithm [12, 13]. The maximum-likelihood (ML) phylogenetic trees were then established based on the VP1 gene by using the ML algorithm in MEGA7 with the bootstrap test of 1000 replications under the best fit models, which were calculated by the tool integrated into MEGA7. The generated tree files were further visualised and edited by the Figtree software. The potential recombination events of the detected EV strains were determined by using the software Recombination Detection Program v4 (RDP4) [14]. And then, the Simplot was used to characterise and visualise the recombination breakpoints [15].

## Results

### Cases and clinical features

From March–May 2019, a total of 264 clinically diagnosed paediatric patients were enrolled along with documentation in terms of clinical features. Afterwards, a total of 249 (94.32%) cases were confirmed in the laboratory using RT-nPCR. Depending on the determined viral agents, we summarised and listed the major clinical characteristics of the patients associated with three predominant viral types (CVA16, CVA6 and CVA10) in Table 1. Generally, some typical symptoms of HFMD, such as skin eruptions on hands and feet, oral herpes and pharyngeal hyperaemia, were present in all patients. Some other symptoms or items vary with different patients. Severe cases, such as those with encephalitis, myocarditis and neonatal sepsis, were not observed.

**Table 1.** The clinical features of the laboratory confirmed HFMD cases in this study

Clinical characteristic^a^	A16 (142)	A10 (10)	A6 (29)
Age（≤ 3）	55 (38.73%)	6 (60%)	23（79.31%）
Gender Ratio (M/F)	1.53	1.5	2.2
Fever (>37.5 °C)	36 (25.35%)	8 (80%)	18(62.06%)
Febrile seizure	2 (1.41%)	0	1 (3.45%)
Skin eruptions on hands and feet	142 (100%)	10 (100%)	28 (100%)
Cough	17 (11.97%)	1 (10%)	1 (3.45%)
Vomit	3 (2.11%)	2 (20%)	1 (3.45%)
Oral herpes	142 (100%)	10 (100%)	29 (100%)
Pharyngeal hyperaemia	142 (100%)	10 (100%)	29 (100%)
Antiadoncus	127 (89.44%)	8 (80%)	24 (82.76%)
Dental ulcer	2 (1.41%)	0	2 (6.90%)
Inappetence	31 (21.83%)	5 (50%)	12 (41.38%)
White blood cell count	8.51 ± 2.31	15.60 ± 8.98	11.77 ± 4.88
Lymphocyte count	2.95 ± 1.00	4.01 ± 1.44	5.13 ± 2.33
Lymphocyte percentage	35.64 ± 11.86	31.86 ± 18.09	44.86 ± 11.67
CRP	5.26 ± 6.94	12.76 ± 13.60	16.3 ± 9.11

a Some clinical features of quantitative value were displayed in the form of mean ± s.d. (range).

### Genetic diversity of EV types involved

**Fig. 1.**
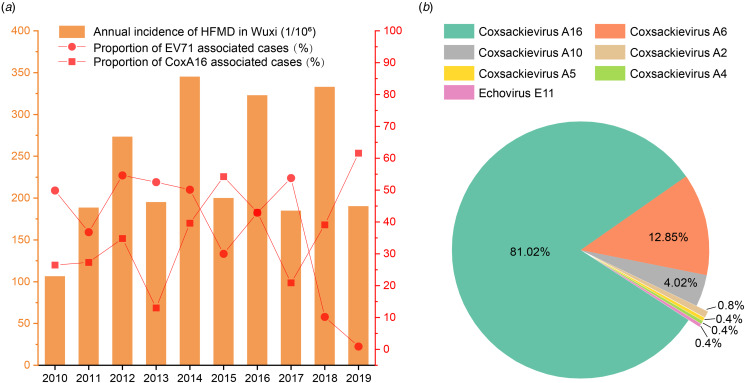
(a) The basic epidemiological information of HFMD in Wuxi during 2010–2019. The annual incidence of each year was indicated by the histogram. The percentage of the EV71 or CVA16 associated cases in the total reported cases were indicated by the dot line; (b) A pie chart representing the percentages of discovered EV strains in Wuxi enrolled patients in 2019.

Depending on the epidemiological data of the HFMD Surveillance System, before 2018, the agents responsible for the HFMD in Wuxi were majorly dominated by the EV71 (46.08%) [9]. As the EV71 vaccination campaign was implemented in 2018, the proportion of EV71 associated cases was reduced to 0.93% in 2019, even though the annual incidence of this disease did not decline (Fig. 1a). On the contrary, the CVA16 became the main reason for the cases after the campaign. Using the RT-nPCR based on degenerate primers for the VP1 gene of different EV species, seven types of EVs belonging to 2 species (Enterovirus A and Enterovirus B) were detected from enrolled paediatric cases in 2019. In accordance with the epidemiological surveillance, the molecular detection results revealed that CVA16 was responsible for the most HFMD cases in 2019 (Fig. 1b). Another major agent was the Coxsackievirus A6 (CVA6), albeit accounting for 12.85% of cases. Some other agents belonging to Enterovirus A, such as Coxsackievirus A10, Coxsackievirus A2, Coxsackievirus A5 were found in few cases. The only one belonging to Enterovirus B was the Echovirus E11 found in 1 case (Fig. 1b). As a result, a wide variety of enteroviral types circulated among children in Wuxi. Despite this, no cases of mixed infection were discovered.

### Phylogenetic and recombination characteristics of circulating EV types in Wuxi

Except for the E11 strain (E11/Wuxi43/China/2019), the full genome sequences for all viral types were recovered. Additionally, the partial sequences of the VP1 gene of all identified strains were determined. The genomic structures of all acquired genomes have been determined and are summarised in Table 2. In addition, all the genome sequences had been submitted to Genbank (Table 2). To determine the phylogenetic relationship of the identified viruses with reported strains, we constructed the ML tree of each EV type based on the VP1 gene. In addition, the potential recombination events of whole-genome sequences were further determined by using the RDP and Simplot packages.

**Table 2.** The molecular features of the EV types detected in Wuxi

Detected Wuxi strains	Blastn hit	Identity	Genome structure					Accession number
			5′UTR	P1	P2	P3	3′UTR	
CVA2/Wuxi261/China/2019	CVA2/Shenzhen21/CHN/2015	97.40%	1–746	747–3323	3324–5057	5058–7319	7320–7387	MZ491028
CVA4/Wuxi145/China/2019	A1/Taian/SD/2018	98.21%	1–743	744–3353	3354–5087	5088–7349	7350–7372	MZ491029
CVA5/Wuxi248/China/2019	CV-A5-3487-M14-XY-CHN-2017	96.68%	1–736	737–3316	3317–5050	5051–7312	7313–7380	MZ491030
CVA6/Wuxi01/China/2019	21/CQ/CHN/2018	98.54%	1–671	672–3281	3282–5015	5016–7277	7278–7346	MZ491031
CVA6/Wuxi17/China/2019	22/HLJ/CHN/2018	98.70%	1–735	736–3346	3347–5080	5081–7341	7342–7413	MZ491032
CVA10/Wuxi83/China/2019	HEV9667699	97.81%	1–743	744–3329	3330–5063	5064–7325	7326–7381	MZ491033
CVA10/Wuxi214/China/2019	R6-19/XY/CHN/2017	97.15%	1–747	748–3333	3334–5067	5968–7329	7330–7373	MZ491034
CVA16/Wuxi35/China/2019	C138/CHW/AUS/2016	97.61%	1–743	744–3329	3330–5063	5064–7325	7326–7382	MZ491035
CVA16/Wuxi108/China/2019	SAX17-50/SaX/Central/CHN/2017-09-16	98.80%	1–718	719–3304	3305–5038	5039–7300	7301–7382	MZ491036
E11/Wuxi43/China/2019	2017-122-R2	96.86%	1–740	741–3323	3324–5057	5058->7019^a^	NA	MZ491037

a Note: The whole genome of the E11/Wuxi43/China/2019 was not finished owing to lacking sequence of some nt in the 3′ terminal. The coding region of the P3 was incomplete.

**Fig. 2.**
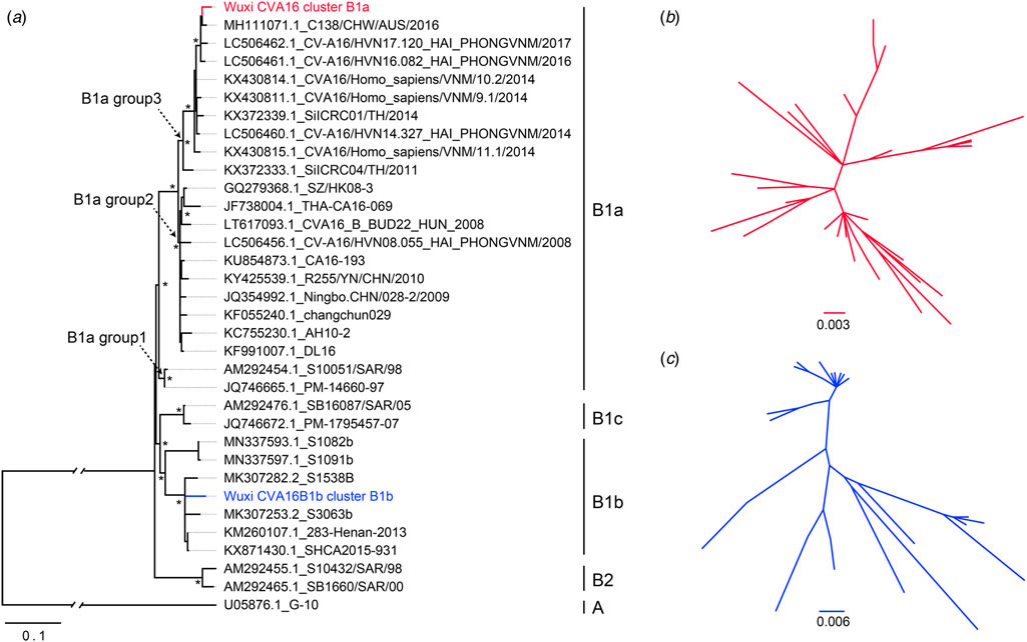
The ML phylogenetic tree based on the VP1 gene of detected CVA16 with reference sequences. (a): The general view of the phylogeny tree. (b and c): independent trees reveal the phylogenetic relationship among all the detected strains of the two viral clusters. The phylogeny trees of b and c were based on the partial VP1 gene.

The phylogenetic analysis revealed that there were two different subtypes of CVA16 circulating in Wuxi (Fig. 2a). A total of 112 strains were assigned to the B1a clade, whereas the remaining strains were assigned to the B1b clade. As the homogeneity analysis based on the whole genome sequence indicated, the strain CVA16/Wuxi35/China/2019, representing the discovered viruses in the B1a clade, shared 97.61% nucleotide identity with C138/CHW/AUS/2016. Phylogenetically, the clade B1a may be divided into 3 groups: 1~3, and the A16 group discovered inside the B1a clade fell into group 3. It is worth noting that all previously reported viruses in group 3 originated in countries other than mainland China, including Australia, Vietnam and Thailand. The Nucleotide identity between the strains CVA16/Wuxi108/China/2019 and SAX17-50/Central/CHN/2017-09-16 was 98.39%. According to the partial VP1 gene-based phylogeny trees, there was some variation in the CVA16 strains, depending on whether they belonged to the B1b or B1a clades (Fig. 2b, 2c).

In 32 patients, two CVA6 subtypes were identified: CVA6/Wuxi01/China/2019 and CVA6/Wuxi17/China/2019. The two subtypes were found together with other strains from different regions of China in the same lineage of the phylogeny tree. The CVA6/Wuxi01/China/2019 was closely related to 21/CQ/CHN/2018 with an identity of 98.54%, and the CVA6/Wuxi17/China/2019 was closely related to 22/HLJ/CHN/2018 with an identity of 98.70%. In 10 patients, two CVA10 subtypes were identified: CVA10/Wuxi83/China/2019 and CVA10/Wuxi214/China/2019, respectively. With a 97.15% identity, the CVA10/Wuxi214/China/2019 was closely related to R6-19/XY/CHN/2017, while the CVA10/Wuxi83/China/2019 was closely related to HEV9667699 with a 97.81% identity. In the phylogenetic tree, the two strains were distinguished as in different clades. Two patients were discovered to be infected with the same strain of CVA2. The identified CVA2 strain, designated CVA2/Wuxi261/China/2019, shared 97.40% nucleotide identity with CVA2/Shenzhen21/CHN/2015, a strain isolated in Shenzhen, Guangzhou province. They were clustered into a unique linage in the phylogenetic tree together with other strains from different regions of China. The detected CVA4 strain, CVA4/Wuxi145/China/2019, was found in one patient and is closely related to A1/Taian/SD/2018, a CVA4 strain detected in Taian city, Shandong Province, with 98.21% nucleotide identity. The clade that the detected CVA4 belonged to consisted of viral strains mainly from China, along with a strain from Australia. The detected CVA5 strain, CVA5/Wuxi248/China/2019 was found in one patient and is closely related to a strain CV-A5-3487-M14-XY-CHN-2017, with 96.68% nucleotide identity.

**Fig. 3.**
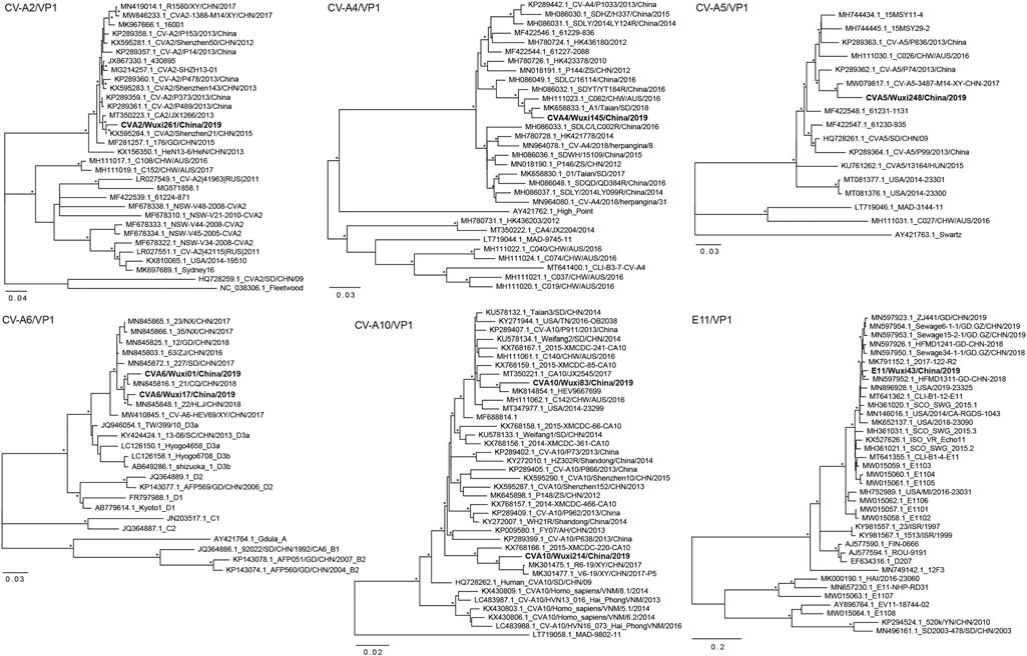
The ML phylogenetic tree based on the partial VP1 gene of detected EV types. Each detected strain was marked as bold type.

The detected E11, named E11/Wuxi43/China/2019, belonged to the species Enterovirus B and was found in one patient. It was closely related to 2017-122-R2 with an identity of 96.86%. The clade of the detected E11 is comprised of various strains in China, the United States of America and the United Kingdom derived from HFMD patients or environmental samples (Fig. 3).

**Fig. 4.**
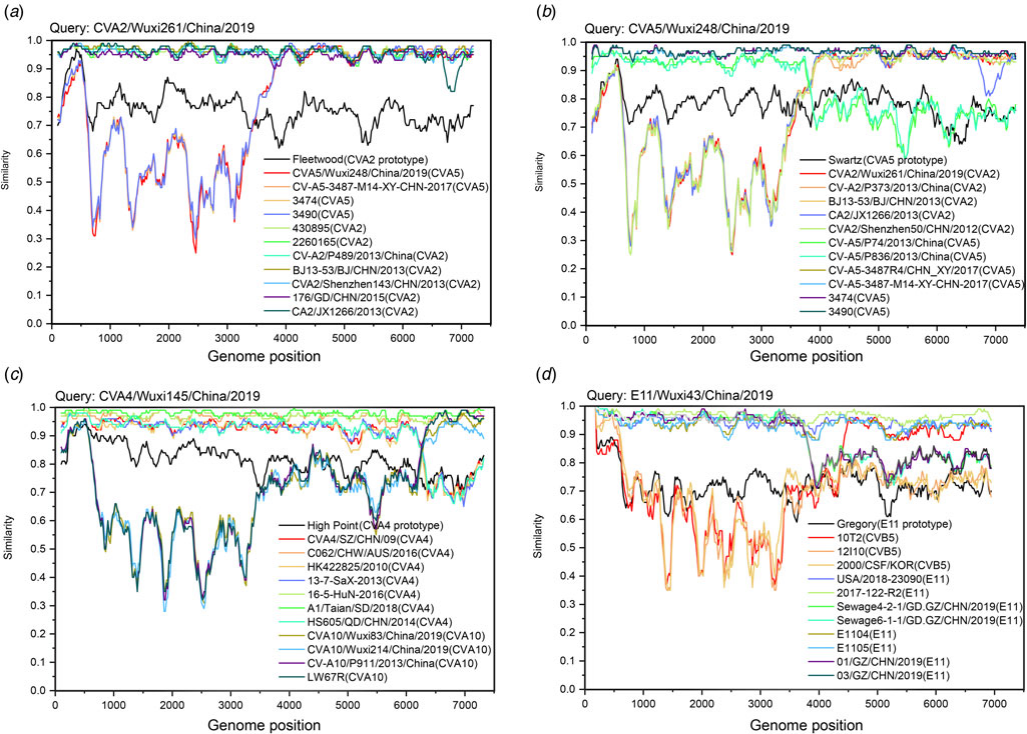
Simplot analysis of various EV strains illustrates the respective recombination events in genomes of CVA2, CVA5, CVA4 and E11 obtained in this study and their reference strains.

As the results of the recombination analysis indicated, recombination occurred between the CVA2/Wuxi261/China/2019 and CVA5/Wuxi248/China/2019 with breakpoints in the P2 region of their genomes (Fig. 4a and 4b). Besides, the CVA4/Wuxi145/China/2019 was observed to have recombination events with potential parental strains of CVA10 in Wuxi (Fig. 4c). In addition, the E11/Wuxi43/China/2019 was discovered to have a mosaic recombinant structure within the region P2, possibly derived from CVB5 (Fig. 4d).

## Discussion

Due to the great diversity and high infectiousness of the corresponding EVs, HFMD was extremely prevalent and difficult to prevent [16]. As an Asia-Pacific country, China endures a significant socioeconomic cost from HFMD [4]. EV71 and CVA16, which mainly affect children under the age of five, have been identified as two primary causal agents for HFMD outbreaks and severe patients in many regions [1]. The introduction of the EV-A71 immunisation in 2016 resulted in a significant decrease in both the number of patients with EV-A71-related HFMD and severe cases, demonstrating the favourable effect of the vaccination campaign. A longitudinal surveillance study conducted in Chengdu, a big city in southwest China, revealed the initial evidence for the effectiveness of the programmatic vaccination against EV71, in line with subsequent studies in some other regions [5, 17, 18]. Except for the decline of the EV71 prevalence, the pathogen spectrum associated with HFMD were also altered as reported in these studies. But the specific situation in different regions varied [6–8]. Wuxi began to promote the EV71 vaccination programme in 2018. Since then, the cases resulting from EV71 had declined distinctly, as expected. Furthermore, the diversity of circulating EV strains in Wuxi has shifted in comparison to the situation ten years ago. In this study, we first identified aetiological traits in multiple molecular aspects of paediatric HFMD cases from a single hospital in Wuxi after programmatic vaccination.

Before 2018, studies based on routine surveillance data revealed that the causal agents for HFMD in Wuxi were dominated by EV71 (46.08%) and CVA16 (35.78%). CVA6, CVA10, Coxsackievirus B5, Coxsackievirus B3, Echovirus 18 and Echovirus 30 were reported in the Non-EV71 and Non-CVA16 HFMD cases in various years with varying incidence rates [9, 10]. While the incidence rate of EV71 had dropped dramatically from 10.18% in 2018 to less than 1% in 2019. On the contrary, since 2018, the incidence rate of CVA16 increased. Some previously circulating agents associated with non-EV71 and non-CVA16 cases were not identified and replaced by others, such as Coxsackievirus A2, Coxsackievirus A4, Coxsackievirus A5 and Echovirus 11.

Similar to EV71, CVA16 was considered the predominant agent responsible for the HFMD outbreaks in many regions of China in the last decade [2, 19–21]. Phylogenetically, there are three genotypes of CV-A16 (Genotypes A, B and D) detected worldwide [19]. Multiple lineages within group B (B1a, B1b and B1c) have been observed co-circulating in Malaysia, Thailand, Australia, Vietnam and Japan. As reported, the circulating A16 strains in mainland China were dispersed in the clades B1a-B1c, with B1b strains accounting for the majority of them [21]. However, in Wuxi, the most circulating CVA16 strains were from B1a. And interestingly, the strains discovered in sub-group 3, within the B1a cluster, were firstly reported. It's worth noting that more than half of the CVA16 patients were over the age of three, implying that CVA16 was responsible for collective outbreaks in schools and other areas where children congregated.

CVA6 was the second dominating type of large population associated with HFMD cases in Wuxi after 2018. As the surveillance data indicated, CVA6 was detected infrequently in the past years, while it occupied 90% of the ‘other’ associated cases in 2017 [10]. It had been reported that CVA6 was associated with increasingly sporadic HFMD outbreaks in some countries [22, 23]. Furthermore, cases associated with this type featured a clinical symptom of onychomadesis sometimes [24, 25].

Along with the normal clinical manifestations of HFMD, CVA10 infections can result in other serious complications such as onychomadesis, hypercapnia, convulsions, central nervous system problems and even death [26]. Additionally, the CVA10 was one of the most frequently detected EVs in the ‘other’ group related to HFMD [27]. Herein, it is essential to pay more attention to the potential CVA10 epidemic in the future. For the homology feature, even though they were out of the same big clade, the two CVA10 sub-types in Wuxi were as distinctive as their positions in the phylogeny tree, indicating the circulating genotypes of multiple evolutionary routes. Despite the fact that CVA2 is thought to be the cause of some sporadic infections, some CVA2 associated severe cases, even deaths, have been reported in recent years. The genotype distribution of CVA2 in China is diversified and extensive [28]. The CVA2 strain in Wuxi is closely related to those in other regions of China, such as Shenzhen, Jiangxi and Hong Kong. It is noticeable that the CVA2/Shenzhen21/CHN/2015, the one detected in Shenzhen and closely relevant to the Wuxi strain, was detected in a severe case [29]. It was also found that the CVA4 was linked to a number of other diseases, like aseptic meningitis, herpangina and viral myocarditis [30, 31]. Similarly, it was believed that the CVA5 has become a significant cause of a range of disorders in recent years, including herpangina, onychomadesis, stomatitis and even acute encephalopathy [32]. The recombination between the A2 and A5 was noteworthy, as similar recombinants in other regions were reported for HFMD epidemics [33].

Despite the fact that some circulating E11 strains have been detected in China in the previous two decades, the potential risk or public health significance has been neglected [34]. The most recent example is that in 2019, several noteworthy outbreaks associated with E11 were discovered in Guangzhou, a southern city in China. Three children died as a result of these outbreaks, which were triggered by a cluster of E11 strains found in patients and environmental samples [35]. The E11 strain E11/Wuxi43/China/2019 found in this study clustered within the same clade with these Guangzhou strains, which indicates the potential risk in the future.

Although the parental strains were not found in Wuxi, the recombination of the detected types of CVA16, CVA6 and CVA10 had been reported in other regions [36–38]. These recombinants were able to maintain an extensive circulation in Wuxi. In contrast, the detected strains with sporadic cases, such as CVA2, CVA5 and CVA4, were all involved in recombination among strains from Wuxi. For recombinant strain E11/Wuxi43/China/2019, the parental origin indicated the circulation of CVB5. Concerning the serotype recording of CVB5 in the surveillance system before 2018, the recombination event probably occurred in Wuxi with a local strain, which deserves further investigation. Furthermore, given the absence of the circulating record of these recombinant strains in Wuxi before 2018, vigilant monitoring is essential due to their potential to cause regional outbreaks or deaths in the future.

The obvious limitation of this study is the finite sampling scale in terms of collection period and case number. Furthermore, no severe cases were collected in this study, resulting in a lack of information for severe case associated agents. Even though a more comprehensive interpretation of the HFMD in Wuxi based on broader sampling and data scale is deserved, the present study still provides the fundamental molecular characteristics of the circulating EV strains, which is beneficial for providing valuable information for protective countermeasures in the future.

## Conclusion

Our study first identified the molecular properties of the circulating viral strains from paediatric cases with HFMD in Wuxi in 2019, such as genomic structure, phylogenetic relationship and recombination events. The agent community has shifted dramatically as a result of the EV71 vaccination, compared to the condition prior to 2018. In accordance with the surveillance data, the molecular detection results of this investigation demonstrated that the causal agents of HFMD were predominantly CVA16 and CVA6. A CVA16 strain from a subgroup of the B1a clade was discovered on the Chinese mainland for the first time. Some recombination events were observed among some of the detected strains in Wuxi. Despite the fact that some of these types have only infected a small population at the moment, attention should be paid to their potential to cause outbreaks or severe cases.
